# Editorial: Special Issue “Addressing New Therapeutic Strategies Using Models”

**DOI:** 10.3390/vetsci10030230

**Published:** 2023-03-17

**Authors:** Ana I. Faustino-Rocha, Paula A. Oliveira

**Affiliations:** 1 Centre for the Research and Technology of Agro-Environmental and Biological Sciences (CITAB), Inov4Agro, 5000-801 Vila Real, Portugal; 2 Department of Zootechnics, School of Sciences and Technology, 7006-554 Évora, Portugal; 3 Comprehensive Health Research Center, 7006-554 Évora, Portugal; 4 Department of Veterinary Sciences, University of Trás-os-Montes and Alto Douro (UTAD), 5000-801 Vila Real, Portugal

A disease model displays pathological processes observed in human or animal diseases. These models are fundamental tools in biomedical research, and they are invaluable for providing new insights into the mechanisms underlying organ function, establishing the pathophysiology of a disease, and testing potential therapeutic approaches. Models have long been used to study several diseases, including cardiovascular, oncologic, metabolic, infectious, and neurological diseases, among others. Animals or cells displaying pathological processes observed in human or animal diseases have been used as models. Moreover, mathematical models are also of great importance to disease modeling. Animal models offer the unique opportunity to investigate the function of genes and pathways and the in vivo effects of drugs, bridging the gap between basic science and the treatment of diseases. The selection of a suitable model system is a crucial step in research design. For disease modeling to be meaningful, a relevant cellular or genetic phenotype must be observed. Without the use of models, both research and clinical practice worldwide would be vastly different today [1,2,3,4,5,6].

The Special Issue “Addressing New Therapeutic Strategies Using Models” aimed to publish original research works or reviews with models of disease, highlighting their importance in the search for new therapeutic strategies. A total of 13 papers, including 2 reviews and 11 original papers, were successfully accepted, providing promising insights into the use of models in the search for new therapeutic strategies for treating several diseases.

Rasteiro et al. [7] addressed the progress in the diagnosis and therapy of urothelial carcinoma in pet animals and compared it to human bladder cancer. In the second review paper, Nascimento-Gonçalves et al. [3] reviewed available rodent colorectal cancer models, their advantages and disadvantages, and their potential in evaluating the effects of several drugs and natural compounds on this type of cancer.

Davis et al. [8] demonstrated that resveratrol treatment did not appear to induce an increased bleeding risk and could improve greyhound dogs’ blood pressure tolerance to severe hemorrhage. Despite this, they did not observe the kidney-protective effects of resveratrol.

Chang et al. [9] demonstrated that imipramine administration may result in the exacerbation of nonalcoholic fatty liver disease, diabetes, diabetic retinopathy, and kidney injury in C57BL6/J mice subjected to a high-fat diet.

Baillou et al. [10] adapted an in vivo method of multiple consecutive but independent intestinal loops in newborn lambs delivered by cesarean section in which endotoxin responsiveness was retained. This new method allowed for the screening of natural yeast fractions for their ability to stimulate immune responses and limit early *Cryptosporidium parvum* development. The model may also be used to investigate host–pathogen interactions and immune responses in a neonatal controlled environment.

Nefedova et al. [11] determined the virucidal activity of Argovit, Triviron, Ecocid, and lauric acid monoglyceride on in vitro (bacteriophage φ6) and in vivo (IB of chickens) models for SARS-CoV-2 infection. These drugs possessed virucidal activity in the small intestine. Based on the obtained results, they also hypothesized that the transmission of the IB virus in chickens occurs not through the respiratory system but through the intestine, where more viral RNA was found.

Caceres et al. [12] found that the ectopic model can be validated as a good and useful model of tumor development in addition to, rather than contrary to, the orthotopic model in breast cancer research.

Lee et al. [13] verified that risperidone might be a therapeutic agent for paraplegia via the attenuation of the damage/death (loss) of spinal motor neurons and neuroinflammation after asphyxial cardiac arrest followed by cardiopulmonary resuscitation.

Paramasivam et al. [14] observed that there were no significant improvements in the locomotion of monkeys on runways following the delayed grafting of nerve segments until one year later.

Lee et al. [15] verified that the combined extracts of *Erigeron annuus* (L.) Pers. and *Brassica oleracea* Var. improved gerbils’ cognitive impairment (a decline in spatial and learning memory).

Lyros et al. [16] observed that the effects of distal mandibular displacement follow a consistent temporal pattern and are statistically significant. They also emphasized the long-term stability of the outcomes, revealing that the mandible does not demonstrate catch-up growth following treatment.

Lee et al. [17] verified that the operating room use of a prefabricated, patient-specific instrumentation protocol on a dog with an antebrachial growth deformity enriched the morphological experience of bone deformities, aided client communication, gained the surgeon’s confidence, maximized surgical precision, minimized surgical wound exposure and anesthetic time, and was intuitive.

Hickman et al. [18] verified that P rats constitute a useful model for studying trichotillomania as they have the advantage of increased genetic variation, which mirrors the human population.

In conclusion, this Special Issue has provided recent updates and important findings. New models have been developed, and several alternative treatments have been demonstrated in different conditions, namely, cancer, infectious diseases, and metabolic, osteoarticular, and neuromuscular disorders.

## Data Availability

The data presented in this study are available on request from the corresponding author.

## References

[1] Alvarado A., Faustino-Rocha A.I., Colaço B., Oliveira P.A. (2017). Experimental mammary carcinogenesis—Rat models. Life Sci..

[2] Nascimento-Gonçalves E., Faustino-Rocha A.I., Seixas F., Ginja M., Colaço B., Ferreira R., Fardilha M., Oliveira P.A. (2018). Modelling human prostate cancer: Rat models. Life Sci..

[3] Nascimento-Gonçalves E., Mendes B., Silva-Reis R., Faustino-Rocha A., Gama A., Oliveira P. (2021). Animal models of colorectal cancer: From spontaneous to genetically engineered models and their applications. Vet. Sci..

[4] Baptista C.V.J., Faustino-Rocha A.I., Oliveira P.A. (2021). Animal models in pharmacology: A brief history awarding the Nobel Prizes for Physiology or Medicine. Pharmacology.

[5] Nascimento-Goncalves E., Seixas F., GIL DA Costa R.M., Pires M.J., Neuparth M.J., Moreira-Goncalves D., Fardilha M., Faustino-Rocha A.I., Colaco B., Ferreira R. (2022). Appraising Animal Models of Prostate Cancer for Translational Research: Future Directions. Anticancer Res..

[6] Faustino-Rocha A.I., Jota-Baptista C., Nascimento-Gonçalves E., Oliveira P.A. (2022). Evolution of models of prostate cancer: Their contribution to current therapies. Anticancer Res..

[7] Rasteiro A.M., Sá e Lemos E., Oliveira P.A., Gil da Costa R.M. (2022). Molecular Markers in Urinary Bladder Cancer: Applications for Diagnosis, Prognosis and Therapy. Vet. Sci..

[8] Davis J., Raisis A.L., Sharp C.R., Cianciolo R.E., Wallis S.C., Ho K.M. (2021). Improved Cardiovascular Tolerance to Hemorrhage after Oral Resveratrol Pretreatment in Dogs. Vet. Sci..

[9] Chang G.-R., Hou P.-H., Wang C.-M., Lin J.-W., Lin W.-L., Lin T.-C., Liao H.-J., Chan C.-H., Wang Y.-C. (2021). Imipramine Accelerates Nonalcoholic Fatty Liver Disease, Renal Impairment, Diabetic Retinopathy, Insulin Resistance, and Urinary Chromium Loss in Obese Mice. Vet. Sci..

[10] Baillou A., Kasal-Hoc N., Barc C., Cognié J., Pinard A., Pezant J., Schulthess J., Peltier-Pain P., Lacroix-Lamandé S., Laurent F. (2021). Establishment of a Newborn Lamb Gut-Loop Model to Evaluate New Methods of Enteric Disease Control and Reduce Experimental Animal Use. Vet. Sci..

[11] Nefedova E., Koptev V., Bobikova A.S., Cherepushkina V., Mironova T., Afonyushkin V., Shkil N., Donchenko N., Kozlova Y., Sigareva N. (2021). The Infectious Bronchitis Coronavirus Pneumonia Model Presenting a Novel Insight for the SARS-CoV-2 Dissemination Route. Vet. Sci..

[12] Caceres S., Alonso-Diez A., Crespo B., Peña L., Illera M.J., Silvan G., de Andres P.J., Illera J.C. (2021). Tumor Growth Progression in Ectopic and Orthotopic Xenografts from Inflammatory Breast Cancer Cell Lines. Vet. Sci..

[13] Lee T.-K., Lee J.-C., Tae H.-J., Kim H.-I., Shin M.C., Ahn J.H., Park J.H., Kim D.W., Hong S., Choi S.Y. (2021). Therapeutic Effects of Risperidone against Spinal Cord Injury in a Rat Model of Asphyxial Cardiac Arrest: A Focus on Body Temperature, Paraplegia, Motor Neuron Damage, and Neuroinflammation. Vet. Sci..

[14] Paramasivam A., Mickymaray S., Jayakumar S., Jeraud M., Perumal P., Alassaf A., Aljabr A.A., Dasarathy S., Rangasamy S.B. (2021). Locomotor Behavior Analysis in Spinal Cord Injured *Macaca radiata* after Predegenerated Peripheral Nerve Grafting—A Preliminary Evidence. Vet. Sci..

[15] Lee T.-K., Hong J., Lee J.-W., Kim S.-S., Sim H., Lee J.-C., Kim D.W., Lim S.S., Kang I.J., Won M.-H. (2021). Ischemia-Induced Cognitive Impairment Is Improved via Remyelination and Restoration of Synaptic Density in the Hippocampus after Treatment with COG-Up^®^ in a Gerbil Model of Ischemic Stroke. Vet. Sci..

[16] Lyros I., Ferdianakis E., Halazonetis D., Lykogeorgos T., Alexiou A., Alexiou K.-E., Georgaki M., Vardas E., Yfanti Z., Tsolakis A.I. (2022). Three-Dimensional Analysis of Posterior Mandibular Displacement in Rats. Vet. Sci..

[17] Lee H.-R., Adam G.O., Kim S.-J. (2022). Application of Patient-Specific Instrumentation in a Dog Model with Antebrachial Growth Deformity Using a 3-D Phantom Bone Model. Vet. Sci..

[18] Hickman D., Prakash A., Bell R. (2022). Predictive Value of Grooming Behavior for Development of Dermatitis in Selectively Bred P Rats as a Model of Trichotillomania Hair Pulling Disorder. Vet. Sci..

